# Irritable bowel syndrome in midlife women: a narrative review

**DOI:** 10.1186/s40695-021-00064-5

**Published:** 2021-05-31

**Authors:** Pei-Lin Yang, Margaret M. Heitkemper, Kendra J. Kamp

**Affiliations:** 1 School of Nursing, National Defense Medical Center, No. 161, Section 6, Minquan E Rd, Neihu District, Taipei, 114 Taiwan; 2 Department of Biobehavioral Nursing and Health Informatics, School of Nursing, University of Washington, Seattle, WA 98195 USA; 3 Division of Gastroenterology, School of Medicine, University of Washington, Seattle, WA 98195 USA

**Keywords:** Midlife, Women, Gastrointestinal symptoms, Irritable bowel syndrome

## Abstract

Midlife women between the ages of 40 and 65 years have reported multiple challenges due to menopausal, developmental, and situational transitions from younger to older adulthood. During the midlife period, many women seek health care for gastrointestinal symptoms and irritable bowel syndrome (IBS). Multiple factors including stress, poor sleep, diet, and physical inactivity may contribute to IBS or gastrointestinal symptoms in midlife women. As such, a comprehensive assessment and treatment approach is needed for midlife women suffering gastrointestinal symptoms. This article reviews the main aspects of the menopausal transition, sex hormonal changes, abdominal and pelvic surgery, psychosocial distress, behavioral factors, and gut microbiome, as well as their relevance on IBS and gastrointestinal symptoms in midlife women. Also, management strategies for IBS in midlife women are discussed. To date, gastrointestinal symptoms during midlife years remain a critical area of women’s health. Additional research is needed to better understand the contributors to gastrointestinal symptoms in this group. Such efforts may provide a new window to refine or develop treatments of gastrointestinal symptoms for midlife women.

## Background

Midlife women between the ages of 40 and 65 typically experience multiple biological, psychological, and social challenges related to the transitions of menopause, development (i.e., re-discovering self) and situation (i.e., changing family relationships, re-balancing work and personal life) [1–3]. This is also a period when many women seek health care for gastrointestinal symptoms including abdominal pain/discomfort, intestinal gas, and alternations in bowel function such as diarrhea and constipation [4–7]. When no organic cause is found, they may be considered to have functional abdominal pain or irritable bowel syndrome (IBS).

IBS is a disorder of gut-brain interaction characterized by abdominal pain and alterations in bowel function including diarrhea and constipation or both. It is diagnosed with the consensus driven Rome IV criteria [8]. In the U.S. and many western countries, the prevalence of IBS is 12–15% and is predominantly diagnosed in women often before the age of 35. A number of factors including differences in pain sensitivity due to both peripheral and central processing mechanisms, co-morbid psychological distress, small intestinal bacterial overgrowth, and motility contribute to this sex difference [9].

**Fig. 1:**
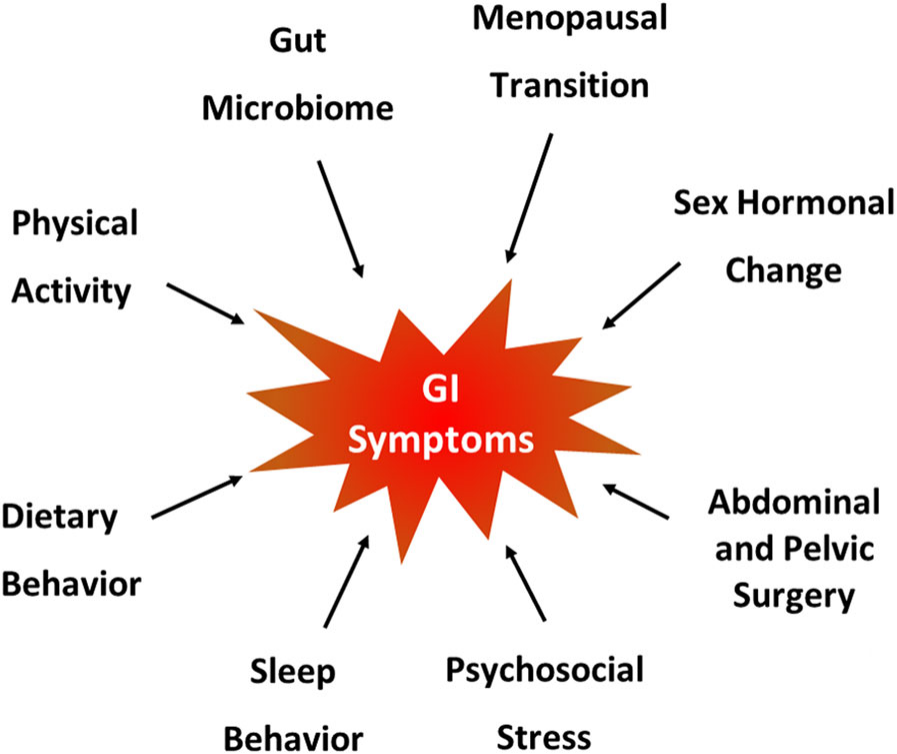
Factors included in this review of irritable bowel syndrome in midlife women

It has long been proposed that sex-related differences in reproductive hormones contribute to clinical phenotypes, in part because sex differences in symptoms and health care seeking behavior emerge at puberty [10, 11]. While both boys and girls with IBS report predominantly constipation, adult women report more constipation and men more diarrhea symptoms. For many women with and without IBS, gastrointestinal symptom severity fluctuates with the menstrual cycle and continues through the menopause transition and into the postmenopausal period [6, 7, 12]. For the most part, little is known about the experience of IBS during the midlife period and/or the menopausal transition. Among women with IBS, cross-sectional studies show either an increase in gastrointestinal symptom severity [4] or no difference when premenopausal women are compared with postmenopausal women [6, 7, 12]. One cross-sectional study reported less severe gastrointestinal symptoms but lower IBS-specific quality of life among midlife and older women with IBS compared to younger individuals [13]. Considering the importance of gastrointestinal health in midlife women, this narrative review addressed several aspects of the menopausal transition and hormonal changes, surgery, stress-related conditions and perceptions, health behaviors (i.e., sleep, hormone use, diet, and physical activity) and gut microbiome (Fig. 1) that may contribute to the experience of IBS and gastrointestinal symptoms in midlife women. Further, we discuss comprehensive strategies for managing functional gastrointestinal disturbances in midlife women as well as future directions in the context of gastrointestinal health in midlife women.

## Methods

We searched PUBMED, CINAHL, EMBASE, and PsycINFO electronic databases up until October 2020 using the following key words: ‘irritable bowel syndrome’, ‘gastrointestinal symptoms’, ‘midlife’, ‘women’, and ‘risk factor’. Publications were eligible for this narrative review if they were written in English and relevant to gastrointestinal health in women. We excluded non-peer review literature, conference abstracts and letters to editor. We identified additional articles from the cited references in the publications retrieved in the first database search. Given this field related to gastrointestinal health in midlife women is developing and that knowledge specifically for midlife women is limited, we conducted a narrative review instead of a systematic review or a scoping review. As such, we did not restrict the inclusion criteria to only midlife women but rather we selected the references based on their relevance to midlife women.

## Factors influencing gastrointestinal health in midlife women

### The menopausal transition and hormonal changes

Midlife is a critical juncture between younger and older adulthood. It is a period of transition during which women experience personal growth balance as well as challenges to emotional and physical health [14]. The menopausal transition is a biological event for midlife women and its median age of onset 47 years and the median age for the final menstrual period is 51.4 years [15]. The menopausal transition is characterized initially by menstrual cycle changes, alterations in estrogen and progesterone levels, and ultimately by cessation of menstruation [16, 17]. Researchers have linked sex hormones with gastrointestinal functions given compelling evidence showing a higher prevalence of IBS or functional dyspepsia symptoms and/or a greater symptom severity in women compared to men [18–22]. Although the underlying mechanisms linking sex hormones and gastrointestinal disturbances remain to be elucidated, intestinal dysmotility, visceral hypersensitivity, intestinal barrier dysfunction and mucosal immune activation through a brain-gut axis are thought to be involved [4, 12, 18, 21, 22]. For example, higher mucosal immune activation was found in women with IBS compared with men with IBS, and mast cell infiltration was related to greater abdominal bloating and dysmotility-like dyspepsia symptoms in women with IBS [23]. A recent longitudinal report on midlife participants in the Study of Women’s Health Across the Nation showed that gut permeability increased from premenopause (followed at a mean age of 49.9 years) to postmenopause (followed at a mean age of 57.5 years). The increase in permeability (i.e., fatty acid binding protein) was associated with a systematic inflammatory marker (i.e., high-sensitivity CRP) [24]. Taken together, future research is warranted to examine whether triggering inflammation during the menopausal transition contributes to gastrointestinal disturbances among midlife women.

To date, it remains to be clarified whether exogenous sex hormone use contributes to or lessens gastrointestinal disturbances among midlife women, particularly those with IBS. When examining the relationship between medication-related sex hormones and symptoms in younger women, Heitkemper et al. [25] found that women with IBS who used oral contraceptives (a mean age of 32.6 years) exhibited reduced monthly abdominal pain symptoms compared to those not using oral contraceptives (a mean age of 32.2 years). Additionally, a review incorporating both animal models as well as younger women suggests pharmacological suppression of ovarian hormones can reduce abdominal pain symptoms [26]. Whether hormone use is linked to symptoms in midlife women was addressed in a population-based study in UK that included over 40,000 women aged 50–69 years with at least one prescription of menopausal hormone treatment (HMT) and 50,000 aged-matched women who never used HMT. Both current (risk ratio [RR]: 1.5, 95% confidence interval [CI]: 1.3–1.9) and past HMT users (RR: 2.0, 95% CI: 1.5–2.6) were at a higher risk of IBS compared to non-users after adjusting for comorbidity and consultation patterns irrespective of treatment duration, regimen or HMT administration route [27]. The discrepancy regarding the effect of sex hormones on gastrointestinal symptoms between these studies could be due to age differences in the populations studied. As such, addressing the influences of fluctuating sex hormones on gastrointestinal motility, immune markers, permeability, and pain sensitivity could inform therapies for midlife women.

Recent efforts to understand the relationship between reproductive hormones and gastrointestinal symptoms have revealed relationships in women without IBS. Callan et al. [7] using annual data collection from the Seattle Midlife Women’s Health Study examined correlates of changes in severity of constipation and diarrhea over 23 years among 291 women during the menopause transition and early postmenopause. Multivariable analyses revealed that neither menopause transition stages (late reproductive, early menopause, late menopause) nor selected sex hormones (estrogen, follicle stimulating hormone and testosterone) were associated with changes in bowel pattern symptom severity over time; however, higher self-reported tension predicted both increases in the severity of constipation and diarrhea, a lower cortisol level predicted an increase in the severity of constipation, and younger age predicted an increase in the severity of diarrhea over time. Recently, Callan et al. [6] assessed abdominal pain from the same dataset. Multivariable analyses showed that younger age, lower estrogen levels and higher anxiety were predictive of an increase in severity of abdominal pain over time, while other sex hormones and menopause transition stages were not. In a cross-sectional study among women with IBS, Lenhart et al. [4] found postmenopausal women with IBS (*n* = 52, a mean age of 54.4 years) experienced significantly worse severity of overall IBS symptoms, physical health-related quality of life and depression than premenopausal women with IBS (*n* = 190, a mean age of 30.2 years), but no differences in abdominal pain, bloating and somatic symptoms were observed. Of note, no difference in the severity of overall IBS symptoms was found between age-matched older and younger males with IBS and thus the authors suggested the potential modulatory effects of estradiol and progesterone on brain-gut interactions.

### Surgical factors

Researchers have considered whether abdominal and pelvic surgery, including hysterectomy and cholecystectomy, may contribute to IBS or gastrointestinal symptoms in adult populations. Hysterectomy is one of the common surgical procedures for women worldwide. It is estimated 20–40% women will experience a hysterectomy by the age of 60 years [28, 29]. Three population-based, cross-sectional studies in the United States (U.S.) [30] and the United Kingdom (U.K.) [31, 32] consistently showed that having a hysterectomy was associated with IBS symptoms (Odds Ratios [ORs]:1.60 ~ 1.80). Numerous population-based, cross-sectional studies in the U.S. [30, 33], the U.K. [31, 34] and Italy [35] also showed that adults with IBS were more likely to report a cholecystectomy history than those without IBS (OR/RRs:1.8 ~ 10.8). Sperber et al. [36] conducted a prospective, case-control study to examine the development of abdominal pain or IBS at 3 and 12 months after elective gynecological surgery for non-painful conditions among 132 women (a mean age of 38.7 years) without pain or bowel symptoms compared with 123 non-surgery controls (a mean age of 35.4 years) without pain or bowel symptoms before surgery. They found higher incidence rates of abdominal pain in women who had undergone surgery compared to the non-surgical controls at both 3 months (10.8 vs. 1.8%, *p* = 0.06) and 12 months (9.0 vs. 1.8%, *p* = 0.02) after gynecological surgery. Psychosocial factors also predicted the development of abdominal pain after gynecological surgery such as an anticipated difficult recovery from the surgery and perceived illness severity. Additionally, as a recent review identified racial, ethnic, and socioeconomic differences in gynecological surgery [37], whether these factors influence risk of developing IBS or gastrointestinal symptoms after gynecological surgery among midlife women is a question that bears additional investigation. To date, the mechanism of post-surgical IBS or gastrointestinal symptoms remain unclear; however, its underlying causes may involve brain-gut dysregulation through a combination of a reduced pain stimulus threshold and pain enhancement as well as perceived psychosocial stress after surgery [38]. Whether midlife women are more vulnerable to post-surgical IBS or gastrointestinal symptoms than women in young or late adulthood remains to be explored in future research.

### Stress-related conditions and perceptions

Murine models of IBS provide evidence that adverse events early in life influence pain-related behaviors in adulthood [39]. Additionally, it is well-established that psychological distress and adverse childhood events are important co-morbid factors associated with IBS [40, 41]. Stress exposure, including adverse early childhood events, is proposed as a key factor accounting for the development and maintenance of pain and disability. For instance, participants with IBS and higher levels of daily psychosocial distress show increased visceral sensitivity to a laboratory oral water loading provocation test compared to those with lower levels of daily psychological distress [42].

For many women, midlife is characterized by multiple co-occurring stressors such as marital and parenting issues, caretaking for children and parents, a work-family imbalance, health problems, deaths, frustrated goal attainment and financial insecurity [1–3, 43, 44]. Such stressful life events make midlife women more vulnerable to psychological distress (i.e., anxiety, depressive mood, stress), irritability and mood swings [45, 46]. Additionally, a review suggests adverse early life experiences can increase susceptibility to psychological distress for women during midlife [47]. For example, a 45-year prospective epidemiological study in a cohort of 9377 British subjects found that at age 45 years, adults who experienced parental physical abuse during childhood (OR: 2.59, 95% CI: 1.94–3.46, *p* < 0.001) and sexual abuse during childhood (OR: 3.37, 95% CI: 2.12–5.34, *p* < 0.001) were more likely to suffer from psychological distress compared to those without adverse childhood experiences regardless of sex and socioeconomic status [48].

The brain-gut axis which bi-directionally communicates between the central nervous system through the autonomic nervous system and the enteric nervous system is a known pathway regulating gastrointestinal function. Thus, stress perceptions can disrupt the brain-gut axis, alter gastrointestinal motility, secretion and visceral sensation, and trigger or exacerbate IBS symptoms [49]. The sympathovagal balance of the autonomic nervous system participates in the regulation of gastrointestinal function including motility, secretion, and gut barrier function can induce anxiety-related hyperalgesia [50, 51]. As shown in meta-analyses, the autonomic nervous system function, as measured by heart rate variability is altered in some adults with IBS and relates to abdominal pain and motility disturbances [52]. In addition, there is some evidence linking autonomic nervous system with gut permeability and inflammation, both thought to contribute to IBS pathophysiology at least in a subset of people [53, 54]. There are both sex- and age-related differences in the autonomic nervous system with both sexes demonstrating a reduction in heart rate variability in middle-aged adults with and without a health condition [55, 56].

Apart from abdominal pain and gastrointestinal symptoms, psychosocial stress plays a key role in non-intestinal pain symptoms in IBS. That is, it is well established that adults with IBS have frequent co-occurring symptoms, e.g., muscle pain, joint pain including temporomandibular/orofacial, headache, and backache [46]. These co-existing pain conditions are more common in women with IBS. When pain-related comorbid symptoms are present, there is an even greater reduction in quality of life, increase in life interference, and increase in health care expenses for those with IBS. Such symptoms tend to group together and may constitute Chronic Overlapping Pain Conditions [57]. Data regarding alterations in central processing of peripheral information are beginning to emerge [58]. Combining data from 5 studies that contained both visceral pain sensitivity and symptom reports, Grinsvall et al. [59] found that when IBS patients were divided into sensitivity tertiles based on laboratory pain/discomfort thresholds, there were significant differences in abdominal pain reports, that is, greater overall symptom severity was associated with increased visceral sensitivity. Increasingly, dysbiosis and ‘leaky gut’ due to psychosocial stress through the brain-gut axis are proposed as mechanisms that may account for the diverse pain-related symptoms in at least a subset of patients with overlapping pain symptoms [57].Psychosocial distress is considered a valuable target for IBS symptom management [60]. Maladaptive behaviors have been associated with IBS symptoms [61, 62]. For example, low resilience has been related to increased IBS symptoms. In a recent U.S national survey, persons with IBS (191 men and 692 women, 85% women, a mean age of 45 years) reported lower resilience as compared to the general population and lower resilience was associated with increased IBS symptom severity. This finding was similar to findings of lower resilience in individuals with other chronic conditions such as inflammatory bowel disease [62] indicating that these characteristics are not unique to IBS and likely related to the chronic, episodic nature of the disorder. Assessments of prior abuse/trauma history, coping behaviors and psychosocial factors may be critical for the treatment of IBS and other chronic gastrointestinal disturbances, particularly for women during midlife.

## Health behavioral factors

### Sleep behavior

Sleep disturbances (e.g., insomnia, poor sleep quality, shorter sleep duration, fragmentation) are well-documented in midlife women transitioning to menopause, affecting 16–42% in premenopause, 39–47% in perimenopause, and 35–60% in postmenopause and are considered a core symptom of menopause [63]. A meta-analysis incorporating 24 cross-sectional studies with a total of 63,542 midlife women showed midlife women at perimenopause, postmenopause or surgical menopause (i.e., menopause induced by surgery) were at higher risks of experiencing sleep disturbances compared to those at the late perimenopausal stage (ORs: 1.60–2.17) [64]. The contributors to sleep disturbance in midlife women are likely complex, that is, age-related changes in sleep architecture, sex hormones (e.g., estrogens), vasomotor symptoms (e.g., hot flashes and night sweats), primary sleep disorders during midlife (e.g., obstructive sleep apnea), medical comorbidities (e.g., hypertension, cardiovascular disease and diabetes), the use of medications and psychosocial (e.g., stress-related conditions and perceptions during midlife transitions) and behavioral factors [16, 65–69].

Left untreated sleep disturbances may lead to numerous physiological alterations such as an imbalance in the autonomic nervous system, activation of the inflammatory pathways, hyperalgesic response to painful stimuli and allodynia, impairment of endogenous pain-inhibitory systems) [70–75]. That, in turn, contributes to IBS symptoms of abdominal pain and co-morbid psychological distress [71, 76]. Recent studies showed that both self-report and objective (i.e., actigraph) reports of poor sleep quality significantly predicted higher levels of next-day IBS symptoms including abdominal pain, and altered bowel patterns in women with IBS at early adulthood [76, 77]. As such, disrupted sleep may be a modifiable risk factor for IBS symptom flare-ups for midlife women [78–80]. Further research is needed to determine the pathophysiological link between poor sleep and gastrointestinal symptoms of midlife women.

Management of sleep disturbances in midlife women can be challenging as multiple contributors to sleep disturbances exist during midlife, and thus require comprehensive sleep assessments and personalized treatments including addressing precipitating and perpetuating factors [67, 81, 82]. The use of low-dose menopausal hormone therapy may be an option in clinical practice to treat menopause symptoms including sleep disturbance [83]. This treatment option is also supported by a recent meta-analysis including 42 randomized clinical trials among midlife women or older at any stage of natural or surgical menopause [84]. However, this meta-analysis did not account for baseline screening of sleep disorders and use of sleep medications. More recently, a population-based study including 13,060 women aged 45 to 75 years from the Norwegian Health Study failed to find a lack of association between menopausal hormone treatment and sleep disturbances when adjusted for relevant covariates, while self-perception of poor health, lifestyle factors (tobacco and alcohol use, less daily physical exercise) and psychological distress (anxiety, depression) were significantly associated with sleep disturbances among midlife women [85]. As such, additional studies are warranted to examine whether these factors relate to gastrointestinal symptoms in midlife women.

Other pharmacological treatments such as non-benzodiazepines zolpidem and eszopiclone are used by clinicians as options to treat insomnia in peri- and early postmenopausal women [65]. Non-pharmacological sleep management such as sleep hygiene approach and cognitive behavioral therapy for insomnia (CBT-I) have also been showed to improve sleep disturbance in midlife women [65, 67]. Kalmbach et al. conducted a randomized control trial (RCT) study on 150 postmenopausal women with chronic insomnia (a mean age of 56.4 years) to compare the effects of CBT-I, sleep restriction therapy in comparison of sleep hygiene education on daytime function, work performance and quality [86]. They found both CBT-I and sleep restriction therapy improved daytime dysfunction (fatigue and daytime sleepiness), quality of life and work performance, and comprehensive CBT-I exhibited superior effects including the added benefit on emotional health improvement in midlife women.

### Dietary behavior

Traditions and stressors related to work and family roles during midlife can shape women’s dietary decisions and behaviors [87, 88]. Many patients with IBS as well as other functional gastrointestinal disorders including functional dyspepsia report their symptoms are triggered by diet; different dietary strategies have been suggested to alleviate symptoms. Although dietary strategies are often perceived as having fewer side effects than medications, dietary modifications may lead to increased cost, inconvenience, or potential unintended consequences [89]. One such unintended consequence is that individuals with IBS may have an underlying eating disorder such as avoidant/restrictive food intake disorder [90]. Therefore, it is recommended that patients be screened for underlying eating disorders prior to engaging in dietary restrictions to reduce gastrointestinal symptom distress [89, 90].

It is well-established that specific foods and drinks are associated with gastrointestinal symptoms. For example, dairy products (e.g., milk, cheese), wheat products, sweets, fried foods, coffee, and alcohol are known to trigger IBS symptoms of abdominal pain, bloating, flatulence and diarrhea [91–95]. Simrén et al. [94] investigated food-related gastrointestinal symptoms among 330 patients with IBS including 243 females and 87 males at midlife, and they found 64% of the sample experienced gastrointestinal symptoms after specific foods and females experienced worse severity of food-related gastrointestinal symptoms. Even though alcohol intake patterns do not vary between women with IBS and healthy controls, alcohol intake patterns significantly relate to gastrointestinal symptoms in women with IBS, while not in healthy controls [95]. In addition to food and drink consumption, eating behavior (eating timing and the regularity of eating pattern) also plays an important role [96, 97]. Guo et al. [96] examined the association between dietary behaviors and IBS in a midlife sample of 78 IBS patients (37 males and 41 females, a mean age of 46.8 years) compared to 79 healthy individuals (44 males and 35 females, a mean age of 43.4 years). They found the individuals with irregular eating habits were more likely to experience IBS symptoms than those with regular eating habits (OR: 3.26, 95%CI: 1.69–6.26, *p* < 0.01). Likewise, an epidemiologic study among 4763 Iranian adults (mean age: 36.5 years) found the sample of women with an irregular eating pattern were at a higher risk of experiencing IBS symptoms compared with those with a regular eating pattern even after adjusting for relevant confounders such as age, BMI, fried food, spice intake and milk intolerance (OR = 1.30; 95% CI:1.02–1.67) [97]. What is unknown is whether irregular eating behaviors during midlife are related to midlife transitions and/or stressors and further contribute to IBS symptoms. Therefore, additional efforts are needed answer this question.

Dietary interventions for IBS have focused on the avoidance of food triggers and use of a low Fermentable Oligo-, Di-, Mono-saccharide And Polyols (FODMAP) diet to reduce symptoms [98, 99]. Currently, the low FODMAP diet is the most commonly recommended diet. A low FODMAP diet involves removing high FODMAP foods from the diet for 2–6 weeks (restriction), gradually reintroducing foods while monitoring symptoms (reintroduction) and developing a personalized plan (individualization) [100]. The diet induces changes in gut bacterial composition including some bacteria involved in the production of intestinal gas and metabolism of bile acids [101]. It is recommended that a low FODMAP diet be conducted with a dietitian in order to oversee dietary restriction and reintroduction. Although adherence to the diet over time is challenging, there do not appear to be adverse nutritional deficiencies linked with the low FODMAP diet [102]. Yet this remains to be determined among midlife women.

### Physical activity

Regular physical activity has been demonstrated to improve gastrointestinal symptoms of abdominal distention and bloating as well as defecation pattern in midlife and older adults through its effects on colonic motility, prompting gastrointestinal transit and increasing abdominal muscle stimulation for defecation [103]. Despite these benefits of physical activity, physical activity declines have been observed in midlife women as they often report difficulties with time as well as multiple roles and responsibilities in family and work [104, 105]. There is still limited information about the effect of physical activity specifically on midlife women and the majority of studies are focused on young adult women with IBS. Lustyk et al. [106] investigated physical activity and symptoms in a cohort of 54 women with IBS and 35 healthy controls (a mean age of 33.0 years). They found women with IBS reported less physical activity than healthy control women. Additionally, physically active women with IBS were less likely to feel incomplete evacuation than physically inactive women with IBS. Johannesson et al. [107] conducted a RCT study on 37 adults with IBS (75.7% women, a median age of 36 years) in a 12-week physical activity intervention group (walking, cycling, swimming) and 38 adults with IBS (73.7% women, a median age of 38.5 years) in a lifestyle maintenance group as control. Compared to controls, adults with IBS in the physical activity group exhibited significantly greater improvement in IBS symptom severity at the 12-week follow-up relative to baseline. Additionally, the investigators [108] followed up adults with IBS in the physical activity group (median follow-up time of 5.2 years) and found long-term benefits of physical activity on improvements of IBS symptom severity, disease-specific quality of life, fatigue and psychological distress at follow-up. In addition to physical activity interventions, yoga has been proposed as a potential therapy for IBS management as yoga-related therapies exhibit greater improvements in IBS symptom severity, psychological distress and health related quality of life than usual care or lifestyle maintenance interventions in RCTs of adults with IBS [109, 110]. In light of these benefits of physical activity and yoga interventions in young adult women, physical activity declines may be a potential modifiable risk factor associated with IBS symptoms in midlife women especially among those with comorbid conditions such as fibromyalgia. However, the benefits of physical activity or yoga on IBS symptoms remains to be evaluated specifically in midlife women.

### Gut microbiome

The gastrointestinal tract is colonized by trillions of bacteria. A recent review has highlighted the interaction between the gut microbiome and immune system homeostasis and activation [111]. Based on evidence that bacterial, viral and parasitic infections can trigger IBS, the role of the gut microbiome in IBS symptoms has moved to the forefront of IBS research [112]. Although some studies of IBS patients support the association of microbiota diversity/composition with IBS abdominal pain, the findings to date have been inconsistent [113, 114]. Sex-specific differences in the gut microbiome (*microgenderome*) are believed to contribute to genetic and epigenetic changes that could potentially lead to systemic immunological changes and ultimately disease states [21, 115]. Animal studies provide evidence the gut microbiome composition changes with aging and that changes are, in part, sex-specific [116].

A challenge in the study of the gut microbiome and symptoms in midlife women is the intersecting role of lifestyle, including diet, medication, and exercise on bacterial composition and metabolic activity. Gastrointestinal symptoms of IBS are intermittent and often wax and wane in terms of severity. Many women with IBS experience an amplification of abdominal pain and looser stools at menses [117]. Although a link between gut microbiome variations across the menstrual cycle or the menopausal transition have not been substantiated, estrogen and the gut microbiome seem to have a bidirectional relationship [118]. Based on findings from a study of anovulatory women compared to ovulatory women, Sasaki reported a greater abundance of *Prevotella*-enriched microbiomes in the anovulatory group [119]. For midlife women, what is unknown is whether the menopausal transition with fluctuations in sex hormones leads to altered gut microbiome and further contribute to gastrointestinal symptoms.

Interventions to influence the gut microbiome have focused on dietary interventions (discussed above), probiotics and fecal microbiota transplant. Although meta-analysis have demonstrated a potentially beneficial influence of probiotics on IBS symptoms [120], a recent technical review from the American Gastroenterology Association has concluded that there is not enough high-quality evidence and therefore probiotics are not recommended [121]. Fecal Microbiota Transplantation (FMT) involves transplanting stool from a healthy donor into an individual with IBS. Preliminary research supports that FMT is an effective treatment for midlife adults with IBS (a mean age of 40 years) including improvements of gastrointestinal symptoms, fatigue and quality of life [122]. Additional research is needed to further advance therapies aimed at influencing the gut microbiome.

## Management of gastrointestinal disturbances in midlife women

The management of gastrointestinal disturbances in midlife women can be challenging because multiple biological, surgical, psychosocial, and behavioral factors may influence gastrointestinal health of midlife women. As such, comprehensive assessments addressing precipitating and perpetuating factors for gastrointestinal disturbances are needed for midlife women. All women should be evaluated for any “red flag symptoms” such as unintentional or unexplained weight loss, rectal bleeding, family history of bowel or ovarian cancer, iron deficiency or an anemia, unexplained bowel habits for more than 6 weeks in people over 50 years old, or elevated inflammatory markers. Also, women aged 45 years are recommended to undergo screening for colorectal cancer [123]. Additionally, given estimated 11.3–18.6% endometriosis prevalence rates in women aged over 40 years [124], healthcare providers should consider the possibility of endometriosis in the midlife women with IBS or gastrointestinal disturbances [125]. Current pharmacologic therapies for gastrointestinal symptoms in people with IBS were developed to affect bowel pattern and/or visceral pain sensitivity. These medications generally include laxatives, smooth muscle relaxants, chloride channel activators, guanylate cyclase agonists, antidepressants, bile acid sequestrants, and antimicrobials. Non-pharmacologic interventions for general IBS populations include education, nutrition counseling, hypnosis, stress reduction, mindfulness training, sleep hygiene, and exercise [60, 126]. Cognitive behavioral therapy (CBT) has received much attention in the IBS literature. CBT is hypothesized to improve patient function by altering dysfunctional cognitions and increasing patient involvement in desirable activities through training in behavioral management techniques such as decreasing positive consequences for illness behaviors, increasing support for wellness behaviors, and use of coping strategies (e.g., relaxation) [100]. Lackner compared a 4-week minimal contact CBT to 10-session clinic-based CBT among 436 adults with IBS (80% females, a mean age of 41 years) and found similar responses with 60–65% of patients considered ‘responders’ (IBS Symptom Severity Score < 50) [127]. But no intervention effectively reduces all symptoms in all people living with IBS.

For adults with IBS, gastrointestinal symptoms usually co-occur (cluster) with non-gastrointestinal symptoms such as psychological distress, sleep disturbance, daytime dysfunction (i.e., fatigue, daily activity and work impairment), and extraintestinal pain symptoms [128]. Of these non-gastrointestinal symptoms, psychological distress [77] and daytime dysfunction [129] have been shown to be value targets for IBS symptom cluster management. As such, a multidimensional treatment approach is suggested for individuals who suffer from gastrointestinal disturbances. For instance, a Comprehensive Self-Management (CSM) Intervention incorporated themes of education, diet (e.g., food composition, trigger foods, meal size or timing), relaxation, and cognitive behavioral therapies [130]. The CSM intervention, delivered in-person or by telephone, has demonstrated significant short-term and long-term effects on improvements of abdominal pain/discomfort, psychological distress, sleep, fatigue and extraintestinal pain symptoms, overall symptom severity, quality of life and daily activity impairment in adults with IBS [130–132]. Adults with IBS can select the most useful strategies for their personalized set of symptoms. Zia et al. [133] followed up a cohort of participants receiving the CSM intervention and found 94% of the participants still used at least 6 strategies, particularly relaxation, diet composition, and identifying thought distortions after 1 year of follow up. Likewise, the effects of combined non-pharmacologic interventions on IBS symptoms are also supported by a recent meta-analysis incorporating 53 RCT studies with various psychological-mind-body therapies such as meditation, relaxation, yoga, autogenic training, progressive relaxation, stress coping, hypnotherapy, and CBT showing medium to high effects of mind-body therapies on IBS symptom severity [60].

Increasingly, behavioral health interventions are being adapted into internet-based digital formats in order to increase acceptability and sustainability. Internet-delivered IBS symptom management interventions have been shown significant improvements in gastrointestinal symptoms and overall symptom severity [126]. As midlife women often report the difficulties with time due to multiple roles and responsibilities, the incorporation of digital technologies into the management may scale efforts to promote treatment adherence for midlife women. Additional investigation is needed to examine whether midlife women would benefit more from digital formatted interventions than traditional ones.

Clinicians caring for midlife women who have persistent gastrointestinal disturbances should consider the diagnosis of IBS. Initial intake can include an assessment of modifiable behavioral factors such as sleep, physical activity, eating behaviors, and diet. Prior to engaging in dietary modification, providers should screen for underlying eating disorders such as avoidant/restrictive food intake disorder. Additionally, screening for history of and current emotional and physical abuse is important for informing and tailoring interventions. Current research is being conducted to determine which biomarkers may predict response to behavioral treatments in order to promote personalized healthcare. However, in the absence of such data, clinicians can develop an individualized plan based on the individuals’ interests. It is important to set realistic expectations that management of gastrointestinal symptoms due to IBS can involve a trial and error approach.

## Conclusion and future directions

Gastrointestinal symptoms during midlife years remain an important unaddressed area of women’s health. Comprehensive assessments for women diagnosed with IBS should address precipitating and perpetuating factors for gastrointestinal symptoms including the menopausal transition, sex hormonal changes, abdominal and pelvic surgery, psychosocial stress, health behavioral factors (i.e., sleep, hormone use, dietary and physical activity) and the gut microbiome. Additional efforts are needed to better understand if these factors contribute to the development of gastrointestinal symptoms among midlife women. Also, it remains unclear whether IBS or gastrointestinal symptoms seen in midlife women are an extension of the IBS or gastrointestinal disturbances of young adulthood, or represent new onset. Midlife women require a multidimensional treatment approach consisting of education, relaxation, and cognitive behavioral therapies for sleep, physical activity and dietary changes such as a low FODMAP diet in order to help them develop personalized treatments. Although the current literature has identified these multiple effective strategies, few studies have specifically focused on the unique needs of midlife women. Additional research focused on midlife women is needed to tailor treatments for midlife women.

## Data Availability

Not applicable.

## Abbreviations

IBS: Irritable bowel syndrome
HMT: Menopausal hormone treatment
RR: Risk ratio
CI: Confidence interval
OR: Odds Ratio
CBT-I: Cognitive behavioral therapy for insomnia
RCT: Randomized control trial
FODMAP: Fermentable Oligo-, Di-, Mono-saccharide And Polyols
FMT: Fecal Microbiota Transplantation
CBT: Cognitive behavioral therapy
CSM: Comprehensive Self-Management
